# Quantitative mismatch between empirical temperature-size rule slopes and predictions based on oxygen limitation

**DOI:** 10.1038/s41598-021-03051-y

**Published:** 2021-12-08

**Authors:** Sigurd Einum, Claus Bech, Øystein Nordeide Kielland

**Affiliations:** 1 Department of Biology, Centre for Biodiversity Dynamics, Norwegian University of Science and Technology (NTNU), Høgskoleringen 5, Realfagbygget, 7491 Trondheim, Norway; 2 Department of Biology, Norwegian University of Science and Technology (NTNU), Høgskoleringen 5, Realfagbygget, 7491 Trondheim, Norway; 3 Norwegian Veterinary Institute, Angelltrøa, PB 4024, 7457 Trondheim, Norway

**Keywords:** Ecology, Physiology

## Abstract

In ectotherms, adult body size commonly declines with increasing environmental temperature, a pattern known as the temperature-size rule. One influential hypothesis explaining this observation is that the challenge of obtaining sufficient oxygen to support metabolism becomes greater with increasing body size, and more so at high temperatures. Yet, previous models based on this hypothesis do not account for phenotypic plasticity in the physiology of organisms that counteracts oxygen limitation at high temperature. Here, we model the predicted strength of the temperature-size response using estimates of how both the oxygen supply and demand is affected by temperature when allowing for phenotypic plasticity in the aquatic ectotherm *Daphnia magna.* Our predictions remain highly inconsistent with empirical temperature-size responses, with the prior being close to one order of magnitude stronger than the latter. These results fail to provide quantitative support for the hypothesis that oxygen limitation drives temperature-size clines in aquatic ectotherms. Future studies into the role of oxygen limitation should address how the strength of the temperature-size response may be shaped by evolution under fluctuating temperature regimes. Finally, our results caution against applying deterministic models based on the oxygen limitation hypothesis when predicting future changes in ectotherm size distributions under climate change.

## Introduction

In ectotherms, adult or maximum body size commonly declines as a plastic response to increasing environmental temperature experienced during their life, a pattern known as the temperature-size rule^1,2^. For size at maturation, this will only result if there is a steeper thermal response in the rate at which maturation is achieved than in growth rate. However, numerous ultimate reasons have been suggested, being either adaptive or due to a constraint, and it is less obvious which one of these that can explain the observed responses^3,4^. One influential hypothesis explaining this observation is that the challenge of obtaining sufficient oxygen to support metabolism becomes greater as body size increases, and that this sets a smaller maximum body size as temperatures and metabolic rates increase^5,6^. Comparative studies provide support for this oxygen limitation hypothesis; the temperature-size relationship (TSR) is relatively strong (i.e. steep slope) in ectotherms that live in aquatic environments, where oxygen availability is relatively low, compared to in their terrestrial counterparts^7–9^. Yet, such evidence is circumstantial, as many features besides oxygen availability differ between aquatic and terrestrial environments in ways that might influence the ecological and evolutionary role of body size, and hence how it may respond to different environmental factors. For example, whereas there is an overall positive correlation between body size and trophic level in aquatic environments, this is not the case in terrestrial environments^10^. Experimental manipulation confirms that exposure to low oxygen levels reduces body size^11^, but this does not necessarily mean that the observed effect of temperature is driven by this response to oxygen limitation. Qualitative evidence for the oxygen limitation hypothesis is provided by experimental studies demonstrating an interactive effect of temperature and oxygen on body size, which show how TSR responses can be more pronounced at low oxygen levels than at normoxia or hyperoxia^12–14^. However, even for such observations, alternative explanations may be envisioned, particularly if oxygen levels in nature are correlated with other ecological factors such as food resource supply or patterns of age-specific mortality, and organisms show adaptive plastic responses in body size to these. Thus, the mechanism behind the temperature-size rule remains an active topic of research^3,4^.

One merit of the oxygen limitation hypothesis is that it allows making quantitative predictions about how strong the temperature response should be. By modelling how the oxygen demand and supply is a function of body size and temperature, the maximum body size that can be sustained under aerobic respiration can be predicted across temperatures. Such attempts should however consider the effect of the phenotypic plasticity organisms can express to counteract oxygen limitations of body size at high temperature. Plastic responses to increased temperature may involve both downregulation of oxygen demand^15,16^ and upregulation of the ability to obtain oxygen from the environment^17,18^. Yet, no studies have predicted the temperature-size response due to oxygen limitation while accounting for the net effect of these two types of plastic responses. One obvious reason for this is that until recently, no estimates of the effect of thermal plasticity in the ability to obtain oxygen on a whole organism level have been available. Thus, previous models have assumed that changes in supply with changes in temperature are determined by the temperature effect on oxygen concentrations^19^, or on the combined effect of temperature on oxygen concentration, viscosity and diffusion rates (i.e. quantified through the oxygen supply index, OSI^20^). Recently, Kielland et al.^21^ provided an empirical estimate of how supply increases with increasing temperature when allowing for phenotypic plasticity for the zooplankton *Daphnia magna*, and demonstrated that this change in supply was insufficient to compensate for the increased demand. Thus, this provided qualitative support for the oxygen limitation hypothesis. Here we apply the data from that study in a model that provides quantitative predictions on how the maximum body size should respond to temperature if the temperature-size rule is driven by oxygen limitation.

## Materials and methods

### Model

At a given temperature *i* there should be a maximum body mass, *Mmax*_*i*_, for which the maximum temperature-dependent surface-specific flux of oxygen, *fmax*_*i*_ (with unit mass O_2_ area^−1^ time^−1^) allows for oxygen uptake to match consumption, and where a further increase in size would lead to an oxygen deficit. This can be expressed as:

1 $$fma{x}_{i}\cdot Ama{x}_{i}={k}_{i}Mma{{x}_{i}}^{\beta },$$

where the left side of the equation gives oxygen uptake and the right side represents oxygen demand. *Amax*_*i*_ is the maximum surface area used for oxygen uptake. Thus, the exact area of the organism that should be considered here will depend on the type of organism (i.e. gill surface area [e.g. fish] or other specific areas of the body surface where oxygen uptake occurs [e.g. ventral body region of *Daphnia*]). *β* is the allometric scaling exponent describing the relationship between body mass and oxygen consumption, and *k*_*i*_ is the parameter describing temperature-dependent oxygen consumption (with unit mass O_2_ body mass^−1^ time^−1^). The relationship between *A* and *M* can be expressed as *A* = *α∙M*^*c*^, where the constant *α* gives the mass specific surface area used for oxygen uptake (with units area mass^−1^) when *M* = 1. The constant *c* is the allometric scaling exponent describing the relationship between body mass and area over which oxygen can diffuse. Thus, since maximum body size will only be limited by oxygen availability when oxygen demand increases faster than supply with increasing body size, the model is only valid for *c* < *β*.

Substituting *Amax* with *α∙Mmax*^*c*^ and rearranging Eq. (1) yields:

2 $$Mma{x}_{i}={\frac{\alpha \cdot fma{x}_{i }}{{k}_{i}}}^{\frac{1}{\beta -c}}.$$

It should be emphasized that *fmax* is the maximum surface specific flux of oxygen which will be reached at the maximum body size. Thus, the surface specific flux of oxygen increases with increasing body size up to *fmax*, which is when oxygen becomes limiting. With this increase in body size, mass specific metabolism decreases with increasing body mass according to the value of the scaling exponent *β.* Thus, systems for delivery of oxygen to cells once it has entered the body will not become constraining with the increasing surface specific flux of oxygen as individual size increases.

By using Eq. (2) on log-scale we can express the linear proportional change in maximum body mass with an increase in temperature from *j* to *i* as:

3 $$l\mathit{og}\left(\frac{Mma{x}_{i}}{Mma{x}_{j}}\right)=\frac{1}{\beta -c}\left(\mathit{log}\left(\frac{fma{x}_{i}}{fma{x}_{j}}\right)-log\left(\frac{{k}_{i}}{{k}_{j}}\right)\right).$$

As can be seen from this, for a given difference between *β* and *c*, the predicted response in maximum body mass to a change in temperature depends on the corresponding relative changes in *fmax* and oxygen consumption. If the proportional change in these two are equal, then no response in maximum body mass is predicted. To evaluate the strength of temperature effects on maximum body mass, Eq. (3) is used to calculate the slope of the change in log maximum body mass with increasing temperature by dividing the right hand size by *i*-*j* (i.e. Δlog *Mmax* °C^−1^). From these slopes, the percentage change per degree increase in temperature is obtained as 100% (e^slope^ − 1).

### Estimating model parameters

For isometric growth, the allometric scaling exponent *c* describing the relationship between body mass and area over which oxygen can diffuse has a value of 2/3. However, many organisms change their body shape throughout ontogeny, resulting in scaling exponents different from 2/3. Using Euclidian geometry, boundary values for this scaling exponent in organisms that lack gills and thus obtain oxygen directly through the body surface can be calculated from the scaling exponent of the body length-mass relationship^22^. For *D. magna* we estimated the scaling exponent of the body length-mass relationship to be 2.72^23,24^, which results in boundary values (possible minimum and maximum values) for the surface area-body mass scaling exponent *c* of 0.684 and 0.735 (see Ref.^22^ for equations). Thus, these values were used in separate calculations of the predicted body mass changes.

Two assumptions are applied to predict body mass changes based on empirical measurements of *fmax*; (1) that the amount of body area available for oxygen uptake for a given body mass, and hence the constant *a*, is independent of temperature, and (2) that *fmax* depends only on temperature and is independent of body size. We describe below how, for our application of the model, assumption (1) can be relaxed, and we also confirm the validity of assumption (2).

Temperature-specific estimates of *k* and *fmax* were obtained using the same approach and data as Kielland et al.^21^, and we repeat the methods of that study in brief here. Individuals of a single clone of *D. magna* were acclimated to 17, 22 and 28 °C over three generations to ensure complete intra- and inter-generational plasticity. *D. magna* from the population used in the study show a monotonic increase in fitness within this temperature range^25^, thus these data conform to the suggested criteria for evaluation of the temperature-size relationship^26^. Measurements of oxygen consumption (*V̇O*_*2*_*)* and critical dissolved oxygen thresholds (*cO*_*2crit*_, i.e. oxygen level above which mass-specific oxygen consumption, *V̇O*_2_*, remains unconstrained, and below which consumption declines) were then conducted on individuals at their respective acclimation temperatures (n = 77, 86 and 84 individuals at 17, 22 and 28 °C, respectively). To provide data on metabolic rates that as closely as possible resemble those experienced in the wild, animals were not starved, and they were allowed to perform their spontaneous swimming activity during measurements. Temperature-specific estimates of *k* were obtained directly from oxygen consumption data (see “Statistics” section). At a given temperature, *fmax* is proportional to the product of how available oxygen is in the environment (i.e. concentration *cO*_2_) and the maximum efficiency by which the animal can obtain it (i.e. maximum area-specific oxygen diffusion into the body per unit oxygen available). The area-specific (and hence mass-specific) oxygen diffusion per unit oxygen available in the environment is at its maximum at *cO*_*2crit*_. Thus, for a given individual, *V̇O*_2_*/*cO*_2*crit*_ provides a measure of the maximum efficiency with which it can obtain oxygen at a given temperature^21^. This measure is identical to what has more recently been termed the oxygen supply capacity (or α)^27^. For each of the three experimental temperatures we multiplied these efficiencies with the corresponding temperature-specific oxygen concentrations at saturation to obtain estimates of temperature-specific values of *fmax*.

The difference in estimated *fmax* across temperatures includes two potential mechanisms. First, there may be effects of temperature on how efficiently individuals obtain oxygen from the environment per area of the body that allows for oxygen uptake. This includes both plasticity in biological characteristics (e.g. oxygen carriers, membrane permeability) and physical characteristics of the water (e.g. diffusivity, viscosity and resulting boundary layers surrounding respiratory surfaces). However, the method used does not allow for quantifying the actual area of the body used for oxygen uptake. Thus, a second effect of temperature on *fmax* in those data may be due to plasticity in the shape of the organism (i.e. proportion of the body surface allowing for oxygen diffusion), and hence the constant *α* in the expression describing the relationship between mass and area given above. Thus, although the model (Eq. (2)) does not explicitly consider potential temperature effects on the relationship between mass and surface area used for oxygen uptake, any such effects are included when using the estimated temperature effects on *fmax* to make predictions about the strength of the temperature-size relationship.

One assumption of our application of the model described above is that *fmax* is independent of body size. This was not tested by Kielland et al.^21^. Thus, we tested for an effect of body mass on *fmax* using their data^28^. We calculated *fmax* for each individual as described above, and fitted an *lme* model (package *nlme*^29^) with *fmax* as a function of temperature (fixed factor) and body mass (mg, covariate), and with run as a random effect. The estimated effect of body mass on *fmax* was weakly negative and non-significant (slope ± SE − 0.29 ± 0.21, p = 0.168). Thus, this assumption appears to be valid for our application of the model.

We also use Eq. (3) to predict the strength of the temperature-size relationship in the absence of phenotypic plasticity. Under this scenario the temperature dependence of maximum oxygen diffusion can be calculated by the OSI approach^20^. According to this, maximum oxygen diffusion will change proportionally with the product of diffusivity and oxygen concentration. Thus,

4 $$fma{x}_{i}\propto OSI\propto D{O}_{2}\cdot p{O}_{2}\cdot \alpha {O}_{2}=D{O}_{2}\cdot c{O}_{2},$$

where *DO*_*2*_ is the diffusivity of oxygen (m^2^ s^−1^, increasing with temperature^20^) and is calculated as a temperature dependent product of viscosity and diffusivity in water^30,31^. *pO*_*2*_ is the ambient oxygen partial pressure, *αO*_2_ is the solubility of oxygen in the water, and *cO*_2_ is the oxygen concentration at saturation (mg O_2_ l^−1^, decreasing with temperature^32^). According to this, OSI increases with increasing temperature^20^. For the experimental temperatures used by Kielland et al*.*^21^, OSI has values of 0.06461, 0.06723 and 0.07037 µg O_2_ h^−1^ m^−1^ at 17, 22 and 28 °C, respectively.

### Statistics

All statistical analyses were carried out in the statistical software R v. 3.3.3^33^*.* We make separate predictions about the temperature-size slope for the two temperature intervals (17–22 and 22–28 °C). To incorporate empirical uncertainty in temperature responses of *k* and *fmax* we used a bootstrapping-procedure to estimate means and 95% confidence intervals (i.e. 2.5 and 97.5 percentiles) for the temperature-size slopes. Each bootstrap replicate was sampled with replacement, with sample sizes equal to the number of observations from each of the 12 runs obtained by Kielland et al*.*^21^. For each replicate sample we calculated *fmax* for each individual based on their *V̇O*_2_*, cO_*2crit*_, and the oxygen content at 100% saturation at their respective temperatures. We then fitted an *lme* model (package *nlme*^29^) with *fmax* as a function of temperature (fixed factor), and with run as a random factor. From this model we extracted the estimated temperature-specific values of *fmax*. We then obtained the temperature-specific oxygen consumption parameter *k* from the same replicate sample using an *lme* model containing log (oxygen consumption) as the dependent variable, temperature as a fixed factor, log (body mass) as a covariate, and run as a random factor. The intercepts from this model (i.e. for a *Daphnia* of 1 mg) were back-transformed and used as estimates of *k*. Finally, all the above parameter estimates were applied together with the estimated allometric scaling exponent^21^ (*β* = 0.801) in Eq. (3) to predict the temperature-size slope for that replicate. A total of 10,000 replicates were run to estimate mean values and 95% confidence intervals. The temperature-size slope predictions were calculated separately for the two boundary values of the surface area-body mass scaling exponents (*c* = 0.684 and 0.735). To produce equivalent estimates of predicted slopes when *fmax* ∝ OSI, this bootstrap procedure was repeated while setting the temperature-specific values of *fmax* equal to the calculated OSI values (see above).

## Results

Both oxygen consumption and critical oxygen thresholds increased with increasing temperature (Table 1). However, the latter increase was considerably smaller than the former, particularly for the upper temperature range. Thus, despite the decline in oxygen content at saturation with increasing temperature, *fmax* increases with increasing temperature (Table 1).

**Table 1:** Estimated parameters from *Daphnia magna* respiration experiment used to predict the strength of the temperature-size relationship.

Parameters		17 °C	22 °C	28 °C	
*V̇O*_2_*		0.0125	0.0144	0.0185	Mass-specific oxygen consumption standardized to mean body size (mg mg^−1^ h^−1^)
*cO*_2*crit*_		1.14	1.20	1.23	Critical oxygen level (mg l^−1^)
*k*		0.0061	0.0069	0.0089	Mass-specific oxygen consumption standardized to 1 mg *Daphnia* (mg mg^−1^ h^−1^)
*β*	0.801				Allometric scaling exponent for body mass—oxygen consumption
c	0.684/0.735				Allometric scaling exponent for surface area—body mass
*fmax*		0.1076	0.1086	0.1237	Maximum mass-specific oxygen uptake (mg mg^−1^ h^−1^)

For temperature-dependent parameters, separate values are given for measurements conducted at different temperatures.

**Figure 1:**
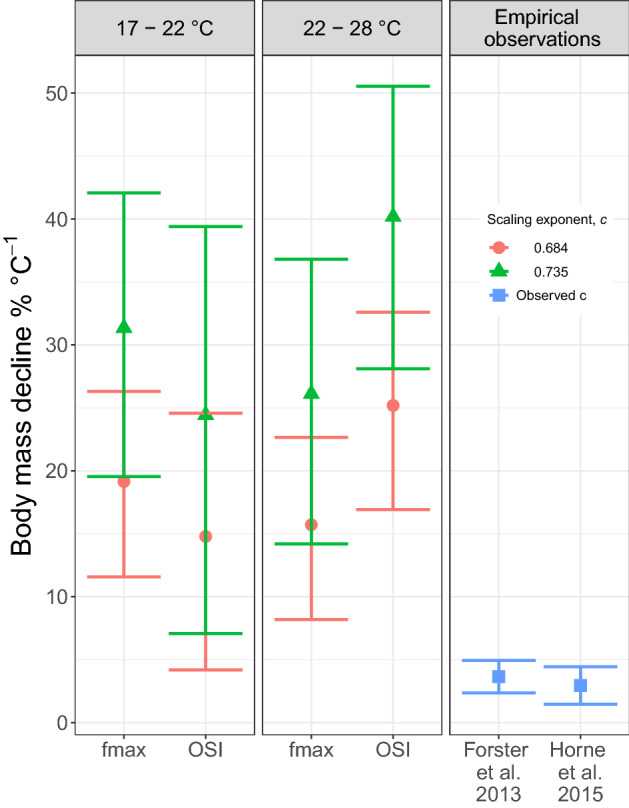
Predicted and observed declines in body mass of aquatic ectotherms with increasing temperature (temperature-size rule slopes, error bars ± 95% CI). “*fmax*” gives the predicted slopes based on empirical measurements of *fmax*, and “*OSI*” those based on the oxygen supply index. Slopes were estimated for two temperature intervals, and for the two boundary values of the surface area-body mass scaling exponent *c* (red circles *c* = 0.684, green triangles *c* = 0.735). Values (mean ± 1.96 SE) from two meta-analyses of aquatic ectotherms are given for comparison.

When applying these estimates to our model (Eq. (3)), body mass was predicted to decline with increasing temperature for both temperature intervals, for both values of the surface area-body mass scaling exponent (*c*), and independent of whether the model allowed for phenotypic plasticity or not (i.e. using *fmax* or OSI) (Fig. 1). Furthermore, none of the confidence intervals overlapped with zero. For the lower temperature interval (17–22 °C) there was little difference in the predicted response of maximum body mass to temperature between the two versions of the model (with or without phenotypic plasticity). For the upper temperature interval (22–28 °C) the mean predicted response was considerably lower when using the empirical estimates of *fmax* than when using OSI. However, all predicted temperature responses, independent of procedures for calculation, greatly exceeded empirical observations (range in mean predicted response was 15–40% decline in mass °C^−1^ depending on temperature interval and value of *c*, mean empirical observations 3.0 and 3.7%, Fig. 1).

## Discussion

The present study evaluates to what extent model predictions, based on the oxygen limitation hypothesis, fit empirical temperature-size slopes when accounting for phenotypic plasticity. In accordance with the temperature-size rule our model predicts declines in maximum body mass with increasing temperature. However, the predicted strength of the response was considerably more pronounced than published empirical temperature-size relationships from aquatic ectotherms^7,8^, including a previously estimated temperature response^25^ in size at maturation over the interval 17–28 °C for the clone of *D. magna* used in the current study (− 1.7% °C^−1^). Admittedly, many of the empirical data used in the meta-analyses^7,8^ are on size at maturation rather than maximum size, and it is the latter that is predicted in our model. However, although temperature responses at these two life stages may differ^34^, the strength of the temperature-size relationship between taxa of aquatic ectotherms does generally not appear to be depend much on the type of data (i.e. Diptera, Ephemeroptera and Odonata that do not grow after reaching maturity vs. the indeterminate growing Crustacea, Fig. 1 in Ref.^8^). It should also be noted that studies in at least one of the meta-analyses was only included if individuals received food at or above saturation across temperatures^8^, and *ad lib* feeding conditions were ensured across temperatures in the study on *D. magna*^25^, such that the strength of relationships was not influenced by nutrient limitations.

Our model predictions incorporate the effects of uncertainty in the estimation of temperature sensitivity of oxygen supply (*fmax*) and demand (*k*). We can also use Eq. (3) to calculate the value for the difference between the scaling exponents *β* and *c* that would be required to predict a temperature response in maximum body mass equal to those observed empirically. We do this for an empirically supported body mass response of 3% °C^−1^, which requires a slope in Eq. (3) of approximately − 0.03. Thus, we have that

$$\beta -c=\frac{-0.03\left(i-j\right)}{\mathit{log}\left(\frac{fma{x}_{i}}{fma{x}_{j}}\right)-log\left(\frac{{k}_{i}}{{k}_{j}}\right)}.$$

Using the mean temperature-specific values of *fmax* and *k* (see “Results” section), the transition from 17 to 22 °C yields a value of *β − c* of 0.83, and that from 22 to 28 °C a value of 0.67*.* The boundary minimum and maximum values for *c* that we used in our model (0.684 and 0.735) assume that animals have a smooth surface, and that it is only the body shape that changes during growth. If the surface increases its fractal dimension during growth (e.g. to increase gas transport) this will lead to an increase in the true value of *c*. Such a bias in predicted values of *c* was observed in a few cases where they could be compared with values of *c* based on directly measured surface areas^22^. We can thus safely assume that *c* in *D. magna* is larger than 0.68. This means that *β* need to be larger than 0.68 + 0.83 to predict a body mass response of 3% °C^−1^ for the interval 17–22 °C, and 0.68 + 0.67 for the interval 22–28 °C. Such values of *β* are clearly unrealistic, and we therefore conclude that our results are robust to parameterization.

We propose three potential reasons for the quantitative discrepancy between our model predictions and empirical data. First, the temperature-size response may be completely unrelated to how oxygen supply and demand changes with temperature. Alternative explanations include how maximum body size is shaped by temperature effects on physiological traits, life history traits, and ecological processes such as food resource supply and mortality rates^3,4^. Second, expressing a temperature-size slope of the strength predicted from our model would require rather extreme levels of plasticity in terms of adult body size. Expressing such pronounced plasticity may entail costs that more than outweighs the benefit of being large at cold temperatures. Furthermore, such extreme effects of temperature on size would likely also require plasticity in other ecological traits such as those related to feeding and predation avoidance, due to the accompanying change in optimal diet and predation risk. Third, our model may fail to capture the way by which oxygen supply and demand shapes the realized maximum body size. Indeed, individuals probably never approach their theoretical maximum body size as set by oxygen limitation, but rather cease growing when reaching a certain smaller size to maintain aerobic scope for activity and reproduction. If animals developing at low temperature decrease their body size relative to the modelled maximum more than those developing at high temperature, this would cause a shallower slope of the temperature-size response relative to our model prediction. This may be a likely response for animals, because they typically evolve under fluctuating temperature regimes. Growing to a maximum size set by oxygen constraints if developing at a low temperature would be maladaptive under natural conditions where subsequent temperature increase is likely. Under this explanation, only individuals developing at high temperatures (relative to their natural range) may approach a size that is limited by oxygen supply (while allowing for sufficient aerobic scope), whereas individuals developing in cold environments should remain further from their limit to allow for future temperature increase. The effect of this would be to produce a temperature response that is weaker than predicted by our model.

Distinguishing between these alternative explanations requires further studies. For example, comparative studies of temperature-size responses in populations or species that have evolved under different levels of temperature fluctuations may shed some light on the third explanation given above. Interestingly, temperature-size relationships in *Daphnia* vary considerably among clones and populations, being either dome shaped^25^, linearly declining^34^ or increasing^35^. Thus, this taxon may provide a useful model system for future studies on the, as of yet, elusive ultimate reason for observed temperature-size relationships in aquatic ectotherms. Nevertheless, by demonstrating a pronounced deviation between predicted responses and empirical observations, our study supports previous arguments^36,37^ against using oxygen limitation models to project future size distributions of aquatic ectotherms in response to climate change. This approach has been used to predict dramatic declines in the body size of marine fishes in response to future climate change^38^. For example, Arctic oceans were projected to show an increase in temperature of 0.2 °C during 2000–2050, and no noticeable change in oxygen content. Yet, maximum body size of fish species currently residing in these waters were predicted to decline by close to 10% over the same period due to the effect of increased oxygen limitation with warming^38^. This is far from observed temperature-size relationships in aquatic ectotherms, which are less than 1% decline in mass per 0.2 °C increase^7,8^. We show that even when accounting for phenotypic plastic responses that increase oxygen supply under high temperatures, the predicted slope of the temperature-size relationship remains too steep to describe empirical data well, and it will be highly misleading to employ such simple deterministic models when predicting future changes in ectotherm size distributions.
